# Neutrophil percentage-to-albumin ratio predicts mortality in bladder cancer patients treated with neoadjuvant chemotherapy followed by radical cystectomy

**DOI:** 10.2144/fsoa-2021-0008

**Published:** 2021-04-20

**Authors:** Matteo Ferro, Dragoş-Florin Babă, Ottavio de Cobelli, Gennaro Musi, Giuseppe Lucarelli, Daniela Terracciano, Angelo Porreca, Gian Maria Busetto, Francesco Del Giudice, Francesco Soria, Paolo Gontero, Francesco Cantiello, Rocco Damiano, Papalia Rocco, Roberto Mario Scarpa, Abdal Rahman Abu Farhan, Riccardo Autorino, Antonio Brescia, Michele Marchioni, Andrea Mari, Andrea Minervini, Nicola Longo, Francesco Chiancone, Sisto Perdona’, Biagio Barone, Pietro De Placido, Michele Catellani, Danilo Bottero, Pasquale Ditonno, Michele Battaglia, Stefania Zamboni, Alessandro Antonelli, Francesco Greco, Giorgio Ivan Russo, Salvatore Smelzo, Rodolfo Hurle, Nicolae Crisan, Matteo Manfredi, Francesco Porpiglia, Felice Crocetto, Carlo Buonerba, Alina Danilesco, Mihai Dorin Vartolomei

**Affiliations:** 1 Department of Urology, European Institute of Oncology, IRCSS, Milan, Italy; 2 Department of Cell & Molecular Biology, University of Medicine and Pharmacy, Targu Mures, Romania; 3 Department of Oncology & Haematology-Oncology, University of Milan, Milan, Italy; 4 Department of Emergency & Organ Transplantation-Urology, Andrology & Kidney Transplantation Unit, University of Bari, Bari, Italy; 5 Department of Translational Medical Sciences, University of Naples Federico II, Naples, Italy; 6 Department of Oncological Urology, Veneto Institute of Oncology IOV, IRCSS, Padua, Italy; 7 Department of Urology, ‘Sapienza’ University of Rome, Policlinico Umberto I Hospital, Rome, Italy; 8 Division of Urology, Department of Surgical Sciences, AOU Citta´ della Salute e della Scienza, Turin School of Medicine, Turin, Italy; 9 Department of Urology, University of Catanzaro, Università ‘Magna Græcia’, Catanzaro, Italy; 10 Department of Urology, Policlinic University Campus of Bio-Medicine of Rome, Rome, Italy; 11 Division of Urology, Virginia Commonwealth University Health System, Richmond, VA 23298, USA; 12 Department of Medical, Oral & Biotechnological Sciences, Gabriele d'Annunzio University of Chieti & Pescara, Chieti, Italy; 13 Department of Experimental and Clinical Medicine, Unit of Oncologic Minimally-Invasive Urology and Andrology, University of Florence, Careggi University Hospital, Florence, Italy; 14 Department of Neurosciences, Human Reproduction & Odontostomatology, University of Naples Federico II, Naples, Italy; 15 Urology Unit, AORN Cardarelli, Naples, Italy; 16 Division of Urology, National Cancer Institute IRCCS Pascale Foundation, Naples, Italy; 17 Department of Clinical Medicine & Surgery, University of Naples Federico II, Naples, Italy; 18 Department of Urology, ASST Spedali Civili di Brescia, Brescia, Italy; 19 Department of Urology, University of Verona, Verona, Italy; 20 Clinic of Urology, Centro Salute Uomo, Bergamo, Italy; 21 Department of Urology, University of Catania, Catania, Italy; 22 Department of Urology, San Raffaele Turro Hospital, San Raffaele University, Milan, Italy; 23 Department of Urology, IRCCS Humanitas Research Hospital, Rozzano, Italy; 24 Department of Urology, University of Medicine & Pharmacy Iuliu Haţieganu, Cluj-Napoca, Romania; 25 Urology Unit - Department of Oncology, School of Medicine, University of Turin, Turin, Italy; 26 Department of Oncology & Hematology, Regional Reference Center for Rare Tumors, University of Naples Federico II, Naples, Italy; 27 National Reference Center for Environmental Health, Zoo-prophylactic Institute of Southern Italy, Portici, Italy; 28 Department of Urology, Medical University of Vienna, Wien, Austria; 29 Cardiovascular Disease and Transplant Institute, Department of Urology, Targu Mures, Romania

**Keywords:** bladder cancer, neoadjuvant chemotherapy, neutrophil percentage-to-albumin ratio, neutrophil-to-lymphocyte ratio, survival

## Abstract

**Aim:**

To investigate the prognostic role of neutrophil percentage-to-albumin ratio (NPAR) in muscle-invasive bladder cancer (MIBC) patients treated with neoadjuvant chemotherapy (NAC) and radical cystectomy (RC).

**Patients & methods:**

213 patients were included.

**Inclusion criteria:**

Nonmetastatic, MIBC (cT2-T4aN0M0), at least three cycles of NAC, undergone RC and with blood count within 30 days before NAC.

**Results:**

Five-years overall survival (OS) with NPAR >18 was 34.06% (95% CI: 18.3–50.5) and 65.37% (95% CI: 52.4–75.6) with NPAR <18. Five years cancer-specific survival (CSS) with NPAR >18 was 42.9% (95% CI: 23.9–60.7) and 74.5% (95% CI: 62.6–83.1) with NPAR <18 (p < 0.001). In multivariable analysis, NPAR increased OS of 1.3 points and CSS of 4.37 points.

**Conclusion:**

High NPAR prior to NAC seems to be a strong predictor of OS and CSS in MIBC patients treated with NAC and RC.

## Lay abstract

This research demonstrated that presence of a high neutrophil percentage-to-albumin ratio in patients with muscle-invasive bladder cancer prior neoadjuvant chemotherapy and radical cystectomy is associated with worse outcomes and reduced overall and cancer-specific survival. Thus, neutrophil percentage-to-albumin ratio could be a useful biological marker in clinical practice to help predict outcomes for patients with muscle-invasive bladder cancer.

Bladder cancer (BC) represents the 10th most common cancer worldwide, with a higher incidence in developed countries, and approximately 80% of tumors being diagnosed in elderly patients (age 65 years or older). Males are three- to four-times more likely to develop the disease than women, but at the time of diagnosis females are more likely to have locally advanced cancer [1,2]. Most BCs are related to exposure to environmental and occupational chemicals, tobacco consumption being the most common risk factor [3,4]. Also, a number of industries such as paint, dye, metal and petroleum processing can be incriminated in developing bladder neoplasia [5].

The most common type of BC is urothelial carcinoma, which occurs in 90% of all cases [6]. The majority of BCs can be diagnosed at an early stage. Although 75% are nonmuscle-invasive tumors at first diagnosis, approximately 78% of patients relapse within 5 years [7,8].

The therapeutic algorithm of urothelial carcinoma depends on the depth of invasion of the neoplasia. The treatment strategy of patients with nonmuscle-invasive cancer is transurethral resection of bladder tumor, often followed by intravesical therapy of bacillus Calmette–Guérin or chemotherapy. The recommended therapy of muscle-invasive BC (MIBC) comprises radical cystectomy (RC) with bilateral pelvic lymphadenectomy combined with chemotherapy [9,10], after cisplatin-based neoadjuvant chemotherapy (NAC), which is associated with an 8% absolute increase in 5-year survival [11]. NAC with platinum-based combination therapy prior to RC with pelvic lymph node dissection (PLND) eradicates micrometastases, improving by a well-established 5% the overall 5-year survival benefit compared with surgery alone [12–15].

Currently available predictive models in patients with BC after RC remain inaccurate and mainly depend on clinicopathological factors. Survival rate at 5 years depends on stage, locoregional extension and distant metastasis [16]. The most important prognostic factor is TMN (T: tumor extension; M: presence of distant metastasis; N: lymphnodal involvement) stage, but also several other blood-based markers could be used as outcome predictors such as neutrophil-to-lymphocyte ratio (NLR), lymphocyte-to-monocyte ratio, platelet-to-lymphocyte ratio, albumin-to-globulin ratio, C-reactive protein-to-albumin ratio, inflammation-based index and modified Glasgow prognostic score [17,18].

NLR is reproducible, inexpensive and easily available. High neutrophil count reflects systemic inflammation, which is linked to protumorigenic effect, while low lymphocyte count indicates poor antitumor immune response of the host. Several studies have shown that elevated NLR leads to poor outcomes for patients with MIBC treated with NAC and RC [19,20].

Recently, neutrophil percentage-to-albumin ratio (NPAR) has been studied as prognostic marker of mortality in cardiogenic shock and its value was more sensitive than neutrophil count and albumin level alone. In pancreatic ductal adenocarcinoma, several studies show a prolonged median survival in patients with lower levels of NAR, which also proved predictive of complete remission to neoadjuvant chemoradiation in rectal cancer [21–23].

The aim of our multicenter study was to investigate the prognostic role of NPAR in patients with MIBC treated with NAC and RC.

## Patients & methods

### Patients selection & data collection

Institutional review board approval at each institution was obtained, with all participating sites providing institutional data sharing agreements prior to the initiation of the study. Inclusion criteria were nonmetastatic, muscle invasive (cT2-T4aN0M0) urothelial carcinoma, received at least three cycles of cisplatin-based NAC, undergone RC with PLND and had blood count data available within 30 days before starting first NAC cycle to calculate NPAR. Patients were excluded if they had <3 cycles of cisplatin-based NAC (14 patients), no PLND performed (three patients) or had variant histology (15 patients). A total of 213 out of 245 patients treated between 1 January 2008 and 31 December 2013 at nine academic institutions met the inclusion criteria. Demographical, clinical, pathological and outcomes data were collected and entered in a computerized database. Data integrity, completeness and quality were ensured through internal and external revisions.

### Data extraction

The neutrophil percentage corresponds to the percentage of neutrophils in white blood cells. The NPAR was calculated as the neutrophil percentage divided by albumin concentration in the same blood samples using the formula: (neutrophil percentage [%] × 100/albumin [g/dl]).

### Management & follow-up

All patients had at least three cycles of cisplatin-based NAC followed by RC and PLND with curative intent. Informed consent was obtained from each patient. Pathological evaluation was carried out according to the tumor node metastasis system of the Union for International Cancer Control and to the 1973 WHO grading classification. All patients were generally followed according to European Association of Urology (EAU) guidelines at that time and institutional protocols. Patients had regular follow-up examinations in the outpatient clinic 3, 6, 12 and 24 months after surgery, and annually thereafter. The clinical follow-up included computed tomography at 6, 12 and 24 months and if clinically indicated. Local recurrences were defined as soft tissue mass ≥2 cm occurring within the field of PLND and cystectomy below the aortic bifurcation. Distant recurrences/metastases were defined as those occurring outside the pelvis. End points were time to death (overall survival [OS]) and death due to BC (cancer-specific survival [CSS]). Cause of death was determined by the treating physician, based on chart review corroborated by death certificates when possible.

### Statistical analysis

Using a receiver operating characteristic curve, we determined that a NPAR cut-off of 18 provided the optimal balance between sensitivity and specificity in our cohort, so patients were classified into a low and high NPAR group (NPAR ≤18 or NPAR >18). Association of NPAR with categorical variables was assessed using χ2 tests; differences in continuous variables were analyzed using Mann–Whitney *U*-test. Kaplan–Meier method was used to estimate disease-free survival, OS and CSS; log-rank tests were applied for pair wise comparison of survival. Univariable and multivariable Cox regression models addressed associations with OS and CSS adjusting for the effects of standard clinical and pathological features. Harrell’s concordance index (C index) was used to measure the ordinal predictive power of the model for disease-free survival (data not shown), OS and CSS. All p-values were two sided, and statistical significance was defined as p < 0.05. Statistical analyses were performed using Stata 11.0 statistical software (Stata Corp., TX, USA).

## Results

### Baseline clinical & pathological features

Median NPAR was 15.45 (interquartile range [IQR]: 12.49–18.88) and ranged from 1.51 to 30.05. A total of 152 (71.4%) patients treated with RC after NAC for MIBC had NPAR <18. There was no difference between this group and patients with NPAR ≥18 in terms of gender, American society of Anesthesiologists (ASA) score, Eastern cooperative oncology group (ECOG) score, preoperative hydronephrosis, hemoglobin, NAC regimen (three vs >3 cycles), histology (pure, nonpure urothelial cancer [UC]), nodes and metastasis status at RC, lymphadenectomy extension, positive nodes and node status after RC, pathological stage, and adjuvant treatment (chemo/radiotherapy). However, there were differences among age (p = 0.004), pre-NAC creatinine (p = 0.001), cholesterol (p = 0.02) and fibrinogen (p = 0.01), BMI (p = 0.001), NLR (p < 0.001), clinical stage (p = 0.03), and nodes removed (p = 0.0007) (Table 1).

**Table 1.** Association of clinical and pathological features with neutrophil percentage-to-albumin ratio in 213 patients treated with radical cystectomy after neoadjuvant chemotherapy for muscle-invasive bladder cancer.

	All cohort	NPAR <18	NPAR ≥18	p-value
Total, n (%)	213	152 (71.4)	61 (28.6)	
Mean age, years (SD)	65.86 (10)	64.55 (8.9)	69.13 (12)	**0.004**
Gender, n (%)				
Males	176 (82.6)	126 (82.9)	50 (82)	0.87
Females	37 (17.4)	26 (17.1)	11 (18)	
ASA score, n (%)				
1	6 (2.8)	4 (2.6)	2 (3.3)	0.69
2	99 (46.5)	67 (44.1)	32 (52.4)	
3	101 (47.4)	76 (50)	25 (41)	
4	7 (3.3)	5 (3.3)	2 (3.3)	
ECOG score, n (%)				
0	121 (56.8)	88 (57.9)	33 (54.1)	0.14
1	69 (32.3)	52 (34.2)	17 (27.9)	
2	13 (6.1)	8 (5.2)	5 (8.2)	
3	4 (2)	1 (0.7)	3 (4.9)	
4	6 (2.8)	3 (2)	3 (4.9)	
Preoperative hydronephrosis, n (%)				
No	165 (77.5)	121 (79.6)	44 (72.1)	0.23
Yes	48 (22.5)	31 (20.4)	17 (27.9)	
Hemoglobin mean (SD)	12.61 (1.96)	12.54 (2)	12.78 (1.8)	0.19
Creatinine mean (SD)	1.17 (0.64)	1.24 (0.72)	1.01 (0.29)	**0.001**
BMI mean (SD)	25.92 (3.68)	26.57 (3.67)	24.29 (3.18)	**0.001**
Cholesterol mean (SD)	202 (38.17)	198.2 (36.96)	211.5 (39.79)	**0.02**
Fibrinogen mean (SD)	3.1 (1.2)	2.93 (1.19)	3.53 (1.17)	**0.01**
NLR mean (SD)	2.77 (1.68)	2.1 (1.03)	4.43 (1.83)	**<0.001**
NAC cycles, n (%)				
=3	148 (69.5)	112 (73.7)	36 (59)	0.051
>3	65 (30.5)	40 (26.3)	25 (41)	
Clinical stage at RC, n (%)				
CIS	4 (1.9)	4 (2.6)	0 (0)	**0.03**
T1	27 (12.7)	21 (13.8)	6 (9.8)	
T2	99 (46.4)	63 (41.5)	36 (59)	
T3	56 (26.3)	47 (30.9)	7 (14.8)	
T4	27 (12.7)	17 (11.2)	10 (16.4)	
Histology, n (%)				
Pure UC	184 (86.4)	129 (84.9)	55 (90.2)	0.3
Nonpure UC	29 (13.6)	23 (15.1)	6 (9.8)	
Node status at RC, n (%)				
N0	193 (90.6)	142 (93.4)	51 (83.6)	0.07
N1	13 (6.3)	6 (4)	7 (11.5)	
N2	7 (3.3)	4 (2.6)	3 (4.9)	
Metastasis status at RC, n (%)				0.79
No	207 (97.2)	148 (97.4)	59 (96.7)	
Yes	6 (2.8)	4 (2.6)	2 (3.3)	
Lymphadenectomy extension				
Standard	171 (80.3)	122 (80.3)	49 (80.3)	0.99
Extended	42 (19.7)	30 (19.7)	12 (19.7)	
Nodes removed	18.21 (9.35)	19.6 (9.1)	14.77 (9.14)	**0.0007**
Positive nodes	1.2 (3.34)	1.32 (3.86)	0.91 (1.34)	0.25
Pathological stage, n (%)				
T0	47 (22.1)	38 (25)	9 (14.8)	0.33
Tis	13 (6.1)	9 (5.9)	4 (6.5)	
Ta	9 (4.2)	4 (2.6)	5 (8.2)	
T1	18 (8.5)	13 (8.5)	5 (8.2)	
T2	42 (19.7)	32 (21.1)	10 (16.4)	
T3	58 (27.2)	38 (25)	20 (32.8)	
T4	26 (12.2)	18 (11.9)	8 (13.1)	
Node status after RC, n (%)				
N0	183 (85.9)	132 (86.8)	51 (83.6)	0.11
N1	9 (4.2)	8 (5.3)	1 (1.7)	
N2	17 (7.9)	11 (7.3)	6 (9.8)	
N3	4 (2)	1 (0.6)	3 (4.9)	
Adjuvant treatment, n (%)				
No	191 (89.7)	135 (88.8)	56 (91.8)	0.64
Chemotherapy	12 (5.6)	10 (6.6)	2 (3.3)	
Radiotherapy	10 (4.7)	7 (4.6)	3 (4.9)	

Boldface values represent statistically significant p < 0.05.

ASA: American society of Anesthesiologists; CIS: Carcinoma *in situ*; ECOG: Eastern cooperative oncology group; NAC: Neoadjuvant chemotherapy; NLR: Neutrophil-to-lymphocyte ratio; NPAR: Neutrophil percentage-to-albumin ratio; RC: Radical cystectomy; SD: Standard deviation; UC: Urothelial cancer.

### Association of NPAR with OS

**Figure 1.**
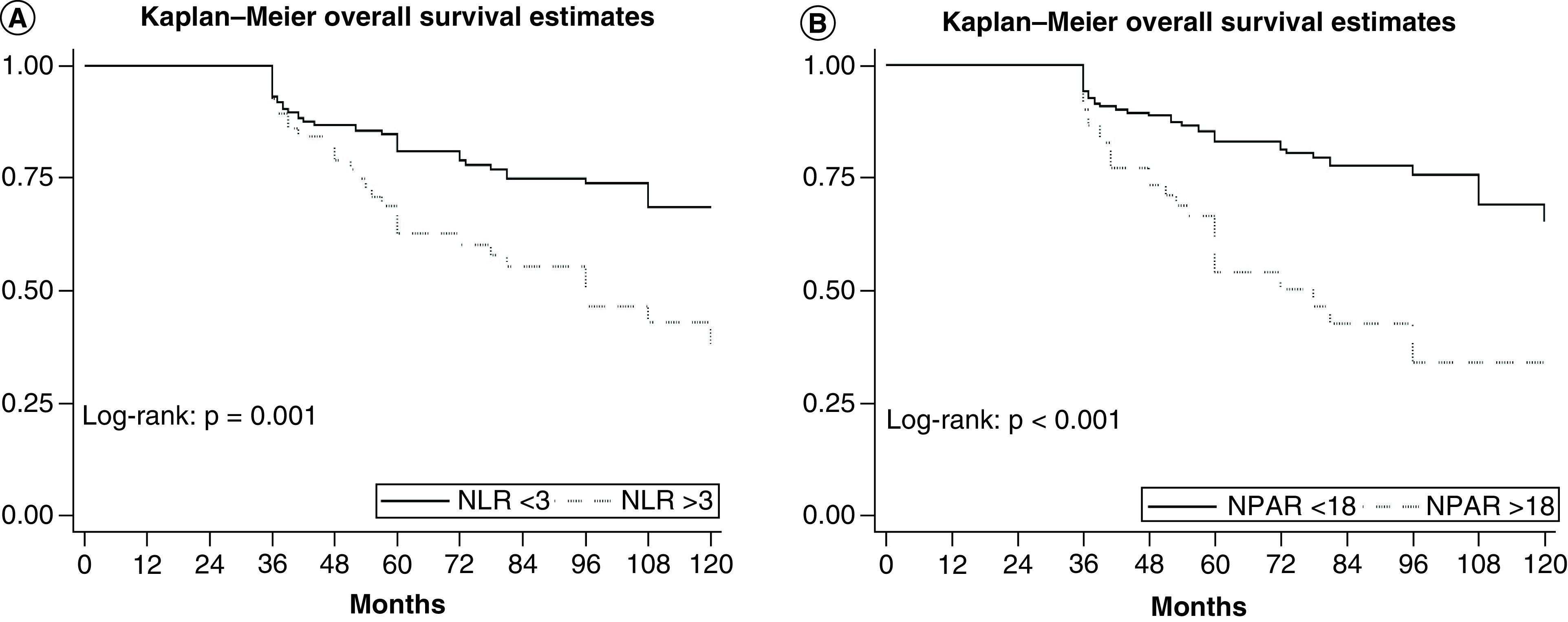
Comparison of overall survival in 213 patients with bladder cancert treated with neoadjuvant chemotherapy followed by radical cystectomy. **(A)** Neutrophil-to-lymphocyte ratio. **(B)** Neutrophil percentage-to-albumin ratio.

During follow-up 65 (30.5%) out of 213 patients died, median follow-up duration was 72 months (IQR: 52–98). Five-years OS estimates among patients with NPAR >18 were 54.2% (95% CI: 38.9–67.2) and 83% (95% CI: 75.9–88.2) in those with NPAR <18, respectively (p < 0.001); and in patients with NLR ≥3 was 62.6% (95% CI: 48.2–74) compared with 80.9% (95% CI: 73.4–86.5) in patients with NLR <3 (p = 0.001) (Figure 1). In univariable analysis, both categorical and continuous NLR (hazard ratio [HR]: 2.17; 95% CI: 1.32–3.54; p = 0.002) and NPAR (HR: 2.9; 95% CI: 1.76–4.78; p < 0.001) were significantly associated with worse OS. In multivariable analysis, independent predictors of OS were patient gender (HR: 1.92; 95% CI: 1.07–3.43; p < 0.02), BMI (HR: 0.88; 95% CI: 0.81–0.95; p < 0.002), hydronephrosis (HR: 1.91; 95% CI: 1.1–3.29; p < 0.02) and tumor stage T4 (HR: 5.44; 95% CI: 2.02–14.66; p < 0.001). Both NLR (model 2, Table 2) and NPAR (model 3, Table 2) retain a statistical significant association with worse OS in multivariable analysis. However, when NLR was added to the predictive model the Harrell’s C index increased only with 0.6 up to 72.1 points, but when we added NPAR instead of NLR the discrimination of the model increased with 1.3 up to 72.8 points (Table 2).

**Table 2.** Preoperative multivariable Cox regression analyses predicting overall survival.

Variables	Model 1			Model 2			Model 3		
	HR	95% CI	p-value	HR	95% CI	p-value	HR	95% CI	p-value
Age cont.	0.98	0.96–1.01	0.29	0.98	0.96–1.01	0.26	0.98	0.95–1.00	0.11
Gender (males vs females)	1.92	1.07–3.43	**0.02**	1.88	1.05–3.36	**0.03**	1.92	1.07–3.44	**0.02**
BMI cont.	0.88	0.81–0.95	**0.002**	0.89	0.82–0.96	**0.005**	0.9	0.83–0.98	**0.01**
Hydronephrosis (no vs yes)	1.91	1.1–3.29	**0.02**	1.62	0.92–2.84	0.09	1.7	0.97–2.96	0.06
Hystology (pure vs nonpure UC)	0.95	0.43–2.06	0.89	0.92	0.42–1.99	0.83	1.08	0.5–2.36	0.83
NAC (3 cycles vs >3 cycles)	1.32	0.78–2.23	0.29	1.3	0.77–2.19	0.31	1.32	0.78–2.22	0.29
Stage, Tis/T1									
T2	1.78	0.7–4.52	0.22	1.44	0.55–3.72	0.45	1.53	0.6–3.9	0.36
T3	1.98	0.73–5.37	0.17	1.85	0.68–5	0.22	1.98	0.74–5.32	0.17
T4	5.44	2.02–14.66	**0.001**	4.9	1.8–13.32	**0.002**	4.83	1.79–13.06	**0.002**
Nodes + (no vs yes)	1.43	0.65–3.1	0.36	1.46	0.67–3.17	0.32	1.26	0.58–2.73	0.54
Metastasis + (no vs yes)	1.12	0.14–8.56	0.9	1.02	0.13–7.83	0.98	1.16	0.15–8.77	0.88
NLR (<3 vs >3)	-	-	-	1.81	1.06–3.09	**0.02**	-	-	-
NPAR (<18 vs >18)	-	-	-	-	-	-	2.28	1.31–3.99	**0.004**
Harrell’s C index	**71.5**			**72.1**			**72.8**		

Boldface values represent statistical significance.

C index: Concordance index; Cont.: Continued; HR: Hazard ratio; NAC: Neoadjuvant chemotherapy; NLR: Neutrophil-to-lymphocyte ratio; NPAR: Neutrophil percentage-to-albumin ratio; UC: Urothelial cancer.

### Association of NPAR with CSS

**Figure 2.**
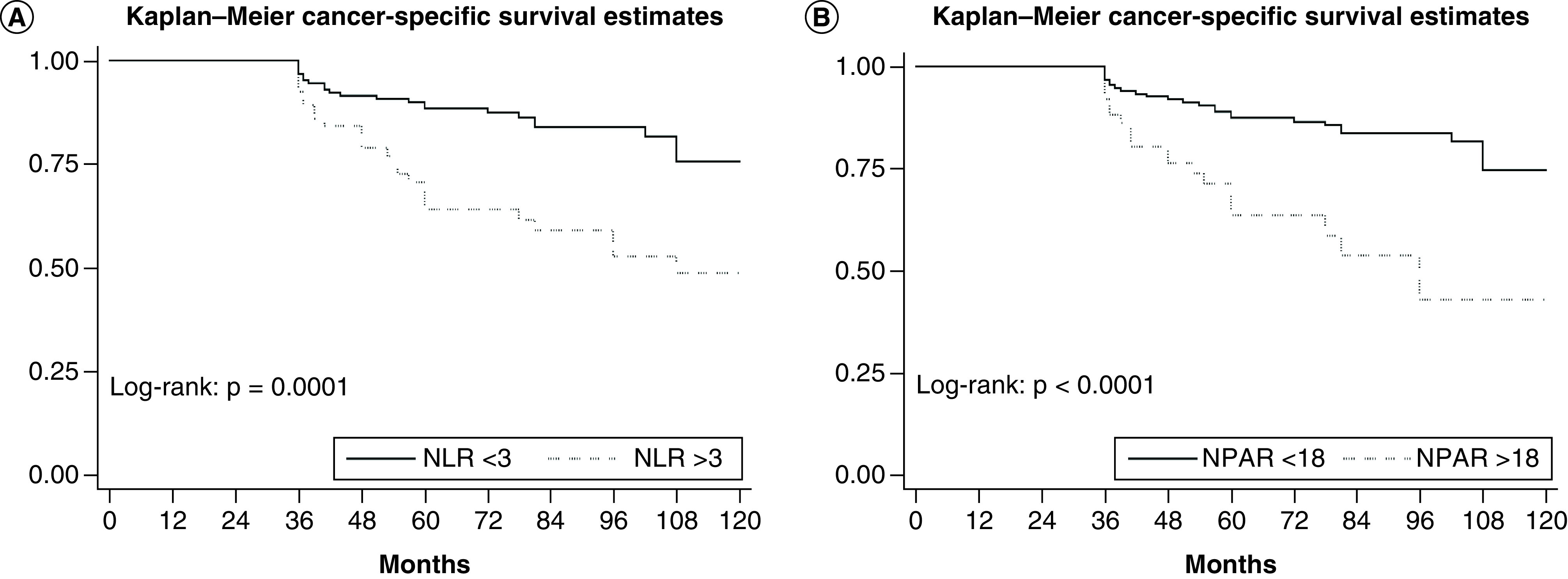
Comparison of cancer-specific survival according to (A) neutrophil-to-lymphocyte ratio and to (B) neutrophil percentage-to-albumin ratio in 213 patients with bladder cancer treated with neoadjuvant chemotherapy followed by radical cystectomy.

During follow-up 48 (22.5%) out of 213 patients died due to BC, median follow-up duration was 72 months (IQR 52–98). Five-years CSS estimates among patients with NPAR >18 were 63.5% (95% CI: 47.8–75.5) and 87.4% (95% CI: 80.7–91.9) in those with NPAR <18, respectively (p < 0.001); and in patients with NLR≥3 was 64.2% (95% CI: 49.8–75.4) compared with 88.3% (95% CI: 81.6–92.7) in patients with NLR <3 (p = 0.001) (Figure 2). In univariable analysis, both categorical and continuous NLR (HR: 2.9; 95% CI: 1.64–5.13; p < 0.001) and NPAR (HR: 3.23; 95% CI: 1.81–5.75; p < 0.001) were significantly associated with worse CSS. In multivariable analysis, independent predictors of CSS were BMI (HR: 0.91; 95% CI: 0.83–0.99; p = 0.04), hydronephrosis (HR: 2.2; 95% CI: 1.08–3.76; p = 0.02) and tumor stage T4 (HR: 4.62; 95% CI: 1.56–13.65; p = 0.006). Both NLR (model 2, Table 3) and NPAR (model 3, Table 3) retain a statistical significant association with worse CSS in multivariable analysis, together with T4 pathological tumor stage. Even more, when NLR was added to the predictive model the Harrell’s C index increased with 3 points up to 70.2, but when we added NPAR instead of NLR the discrimination of the model increased with 4.37 up to 71.57 points (Table 3).

**Table 3.** Preoperative multivariable Cox regression analyses predicting cancer-specific survival.

Variables	Model 1			Model 2			Model 3		
	HR	95% CI	p-value	HR	95% CI	p-value	HR	95% CI	p-value
Age cont.	0.98	0.95–1.01	0.41	0.98	0.95–1.01	0.37	0.98	0.95–1.00	0.18
Gender (males vs females)	1.48	0.72–3.03	0.27	1.44	0.7–2.95	0.31	1.45	0.7–2.98	0.31
BMI cont.	0.91	0.83–0.99	**0.04**	0.92	0.84–1.01	0.09	0.94	0.86–1.03	0.2
Hydronephrosis (no vs yes)	2.02	1.08–3.76	**0.02**	1.6	0.85–3.02	0.14	1.75	0.93–3.31	0.08
Hystology (pure vs nonpure UC)	0.76	0.29–1.99	0.58	0.72	0.27–1.89	0.5	0.88	0.33–2.31	0.8
NAC (3 cycles vs >3 cycles)	1.13	0.6–2.12	0.68	1.12	0.6–2.09	0.7	1.13	0.6–2.11	0.69
Stage, Tis/T1	Ref.			Ref.			Ref.		
T2	1.35	0.48–3.82	0.56	1	0.34–2.88	0.99	1.18	0.41–3.36	0.74
T3	1.77	0.6–5.24	0.29	1.58	0.54–4.7	0.39	1.84	0.62–5.42	0.26
T4	4.62	1.56–13.65	**0.006**	3.84	1.27–11.59	**0.01**	3.89	1.3–11.67	**0.01**
Nodes + (no vs yes)	0.99	0.34–2.87	0.99	1.03	0.35–2.97	0.95	0.85	0.29–2.45	0.76
Metastasis + (no vs yes)	1.44	0.18–11.21	0.72	1.29	0.16–10.12	0.8	1.44	0.18–11.19	0.72
NLR (<3 vs >3)	-	-	-	2.62	1.43–4.79	**0.002**	-	-	-
NPAR (<18 vs >18)	-	-	-	-	-	-	2.82	1.48–5.36	**0.002**
Harrell’s C index	**67.2**			**70.2**			**71.57**		

Boldface values represent statistical significance.

C index: Concordance index; HR: Hazard ratio; NAC: Neoadjuvant chemotherapy; NLR: Neutrophil-to-lymphocyte ratio; NPAR: Neutrophil percentage-to-albumin ratio; Ref.: Reference; UC: Urothelial cancer.

## Discussion

We showed that elevated level of NPAR was associated with worse oncological outcomes in patients with MIBC treated with NAC followed by RC with PLND. Furthermore, when we added NPAR in a preoperative predictive model in both OS and CSS increased the discrimination compared with addition of NLR.

According to our findings, it seems that as was shown in the case of patients with pancreatic cancer and rectal cancer [22,24], NPAR could be a useful biomarker for predicting outcomes in patients with MIBC. Even in nonneoplastic conditions elevated NPAR was related with increased risk of all-cause mortality [25], and elevated admission NPAR was linked to higher rate of death during hospitalization in patients with ST-elevation myocardial infarction [26]. Other studies demonstrated that high NPAR in patients with coronary artery disease and patients with acute kidney injury was correlated with 30-, 90- and 365-day all-cause-mortality [27,28]. Also, an increased level of NPAR was associated with high mortality rate in patients with cardiogenic shock [21].

In a recent multicentric study NLR with a cut-off of three seems to be an important prognostic factor, correlated to disease recurrence, progression and CSS in patients with primary T1 HG/G3 non-muscle invasive bladder cancer (NMIBC) treated with intravesical bacillus Calmette–Guérin therapy [29].

The limitations of our multicentric study should also be recognized. The retrospective design of the study introduces limitations such as the absence of data on potential confounding factors, differential losses to follow-up and information bias [20]. It also may lead to an inaccurate recall, making it difficult to distinguish the exposed and the nonexposed individuals, in order to take drastic conclusions. Second, histology specimens were not examined by central pathology and major risk factors, lymphovascular invasion and variant histology not being evaluated. Also, the patients’ comorbidities could have influenced the decision related to undergoing instillation therapy or surgery, resulting in exclusion from our study. In the end, we have to mention that our NPAR cutoff of 18 was not studied before regarding the prognostic character in patients with invasive bladder neoplasia, as our intent was to study the validity of biomarker at this cut-off value. In fact, negative results of NPAR prognostic factor might not be published, causing potential publication bias.

## Conclusion

By using a cutoff of 18, NPAR appears to be a clinically significant predictor of worse oncologic outcomes in patients with MIBC treated with NAC and RC. Even more, it increases the discrimination in predicting both OS and CSS in comparison with NLR ratio.

## Future perspective

The role of NPAR as prognostic factor in MIBC is quickly growing. Although further studies are required in order to evaluate the weight of this parameter and maximize its utility, the possibility to use an inexpensive and easily available hematologic test to predict OS and CSS of patients with MIBC could be particularly relevant in the future clinical practice.

**Summary points**

- Bladder cancer (BC) represents the 10th most common cancer worldwide, with 78% of patients relapsing within 5 years from conservative treatment (transurethral resection of bladder tumor).
- The recommended therapy for muscle-invasive BC (MIBC) comprises radical cystectomy (RC) with bilateral pelvic lymphadenectomy combined with chemotherapy.
- Currently available predictive models in patients with BC after RC remain poor and inaccurate.
- Neutrophil percentage-to-albumin ratio (NPAR) corresponds to the percentage of neutrophils in white blood cells and is calculated as the neutrophil percentage divided by albumin concentration in the same blood sample using the formula: (neutrophil percentage [%] × 100/albumin [g/dl]).
- Of the 213 patients included, 5-years overall survival estimates among patients with NPAR >18 were 54.2% (95% CI: 38.9–67.2) and 83% (95% CI: 75.9–88.2) in those with NPAR <18 (p < 0.001).
- Similarly, 5-years cancer-specific survival estimates among patients with NPAR >18 were 63.5% (95% CI: 47.8–75.5) and 87.4% (95% CI: 80.7–91.9) in those with NPAR <18 (p < 0.001).
- Elevated level of NPAR was associated with worse oncological outcomes in patients with MIBC treated with neoadjuvant chemotherapy followed by RC with pelvic lymph node dissection.
- NPAR appears to be a clinical significant predictor of worse oncologic outcomes in patients with MIBC treated with neoadjuvant chemotherapy and RC both on overall and cancer-specific survival.

## References

[1] Saginala K, Barsouk A, Aluru JS, Rawla P, Padala SA, Barsouk A. Epidemiology of bladder cancer. Med.Sci. (Basel) 8(1), 15 (2020). 10.3390/medsci8010015 PMC7151633 32183076

[2] Scosyrev E, Noyes K, Feng C, Messing E. Sex and racial differences in bladder cancer presentation and mortality in the US. Cancer 115(1), 68–74 (2009). 19072984 10.1002/cncr.23986

[3] Freedman ND, Silverman DT, Hollenbeck AR, Schatzkin A, Abnet CC. Association between smoking and risk of bladder cancer among men and women. JAMA 306(7), 737–745 (2011). 21846855 10.1001/jama.2011.1142 PMC3441175

[4] Tarantino G, Crocetto F, Di Vito C Association of NAFLD and insulin resistance with non metastatic bladder cancer patients: a cross-sectional retrospective study. J. Clin. Med. 10(2), 346 (2021). 33477579 10.3390/jcm10020346 PMC7831331

[5] Burger M, Catto JW, Dalbagni G Epidemiology and risk factors of urothelial bladder cancer. Eur. Urol. 63(2), 234–241 (2013). 22877502 10.1016/j.eururo.2012.07.033

[6] Alifrangis C, McGovern U, Freeman A, Powles T, Linch M. Molecular and histopathology directed therapy for advanced bladder cancer. Nat. Rev. Urol. 16(8), 465–483 (2019). 31289379 10.1038/s41585-019-0208-0

[7] Pfannstiel C, Strissel PL, Chiappinelli KB The tumor immune microenvironment drives a prognostic relevance that correlates with bladder cancer subtypes. Cancer Immunol. Res. 7(6), 923–938 (2019). 30988029 10.1158/2326-6066.CIR-18-0758

[8] Yuksel OH, Akan S, Urkmez A, Yildirim C, Sahin A, Verit A. Preoperative Glasgow prognostic score as a predictor of primary bladder cancer recurrence. Mol. Clin. Oncol. 5(1), 201–206 (2016). 27330798 10.3892/mco.2016.901 PMC4906850

[9] DeGeorge KC, Holt HR, Hodges SC. Bladder cancer: diagnosis and treatment. Am. Fam. Physician 96(8), 507–514 (2017). 29094888

[10] Bada M, De Concilio B, Crocetto F Laparoscopic radical cystectomy with extracorporeal urinary diversion: an Italian single-center experience with 10-year outcomes. Minerva Urol. Nefrol. 72(5), 641–643 (2020). 32550634 10.23736/S0393-2249.20.03850-3

[11] Yin M, Joshi M, Meijer RP Neoadjuvant chemotherapy for muscle-invasive bladder cancer: a systematic review and two-step meta-analysis. Oncologist 21(6), 708–715 (2016). 27053504 10.1634/theoncologist.2015-0440 PMC4912364

[12] Sherif A, Holmberg L, Rintala E Neoadjuvant cisplatinum based combination chemotherapy in patients with invasive bladder cancer: a combined analysis of two Nordic studies. Eur. Urol. 45(3), 297–303 (2004). 15036674 10.1016/j.eururo.2003.09.019

[13] Advanced bladder cancer (ABC) meta-analysis collaboration. Neoadjuvant chemotherapy in invasive bladder cancer: update of a systematic review and meta-analysis of individual patient data. Eur. Urol. 48(2), 202–205; discussion 205–206 (2005). 15939524 10.1016/j.eururo.2005.04.006

[14] Rosenblatt R, Sherif A, Rintala E Pathologic downstaging is a surrogate marker for efficacy and increased survival following neoadjuvant chemotherapy and radical cystectomy for muscle-invasive urothelial bladder cancer. Eur. Urol. 61(6), 1229–1238 (2012). 22189383 10.1016/j.eururo.2011.12.010

[15] Witjes JA, Bruins HM, Cathomas R European Association of Urology guidelines on muscle-invasive and metastatic bladder cancer: summary of the 2020 guidelines. Eur. Urol. 79(1), 82–104 (2021). 32360052 10.1016/j.eururo.2020.03.055

[16] DeSantis CE, Lin CC, Mariotto AB Cancer treatment and survivorship statistics, 2014. CA Cancer J. Clin. 64(4), 252–271 (2014). 24890451 10.3322/caac.21235

[17] Peng D, Zhang C-J, Gong Y-Q Prognostic significance of HALP (hemoglobin, albumin, lymphocyte and platelet) in patients with bladder cancer after radical cystectomy. Sci. Rep. 8(1), 1–9 (2018). 29335609 10.1038/s41598-018-19146-y PMC5768698

[18] Zhang L, Li L, Liu J Meta-analysis of multiple hematological biomarkers as prognostic predictors of survival in bladder cancer. Medicine 99(30), e20920 (2020). 32791672 10.1097/MD.0000000000020920 PMC7387011

[19] Black AJ, Zargar H, Zargar-Shoshtari K The prognostic value of the neutrophil-to-lymphocyte ratio in patients with muscle-invasive bladder cancer treated with neoadjuvant chemotherapy and radical cystectomy. : Urologic Oncology: Seminars and Original Investigations. Elsevier, 3.e17–13.e27 (2020). 10.1016/j.urolonc.2019.09.023 31676278

[20] Kaiser J, Li H, North SA The prognostic role of the change in neutrophil-to-lymphocyte ratio during neoadjuvant chemotherapy in patients with muscle-invasive bladder cancer: a retrospective, multi-institutional study. Bladder Cancer 4(2), 185–194 (2018). 29732389 10.3233/BLC-170133 PMC5929304

[21] Peng Y, Xue Y, Wang J Association between neutrophil-to-albumin ratio and mortality in patients with cardiogenic shock: a retrospective cohort study. BMJ Open 10(10), e039860 (2020). 10.1136/bmjopen-2020-039860 PMC7574943 33077569

[22] Tingle SJ, Severs GR, Goodfellow M, Moir JA, White SA. NARCA: a novel prognostic scoring system using neutrophil-albumin ratio and Ca19-9 to predict overall survival in palliative pancreatic cancer. J. Surg. Oncol. 118(4), 680–686 (2018). 30196571 10.1002/jso.25209

[23] Li R, Sun Z, Song S NARFIB: a novel prognostic score based on the neutrophil-to-albumin ratio and fibrinogen can predict the prognosis of gastrointestinal stromal tumors. Cancer Manag. Res. 12, 11183–11190 (2020). 33177869 10.2147/CMAR.S281375 PMC7650032

[24] Tawfik B, Mokdad AA, Patel PM, Li HC, Huerta S. The neutrophil to albumin ratio as a predictor of pathological complete response in rectal cancer patients following neoadjuvant chemoradiation. Anticancer Drugs 27(9), 879–883 (2016). 27434664 10.1097/CAD.0000000000000411

[25] Gong Y, Li D, Cheng B, Ying B, Wang B. Increased neutrophil percentage-to-albumin ratio is associated with all-cause mortality in patients with severe sepsis or septic shock. Epidemiol. Infect. 148, e87–e87 (2020). 32238212 10.1017/S0950268820000771 PMC7189348

[26] Cui H, Ding X, Li W, Chen H, Li H. The neutrophil percentage to albumin ratio as a new predictor of in-hospital mortality in patients with ST-segment elevation myocardial infarction. Med. Sci. Monit. 25, 7845–7852 (2019). 31628741 10.12659/MSM.917987 PMC6820334

[27] Sun T, Shen H, Guo Q Association between neutrophil percentage-to-albumin ratio and all-cause mortality in critically ill patients with coronary artery disease. Biomed. Res. Int. 2020, 8137576 (2020). 32934964 10.1155/2020/8137576 PMC7479485

[28] Wang B, Li D, Cheng B, Ying B, Gong Y. The neutrophil percentage-to-albumin ratio is associated with all-cause mortality in critically ill patients with acute kidney injury. Biomed. Res. Int. 2020, 5687672 (2020). 32219136 10.1155/2020/5687672 PMC7049452

[29] Vartolomei MD, Ferro M, Cantiello F Validation of neutrophil-to-lymphocyte ratio in a multi-institutional cohort of patients with T1G3 non-muscle-invasive bladder cancer. Clin. Genitourin. Cancer 16(6), 445–452 (2018). 30077463 10.1016/j.clgc.2018.07.003

